# Synthesis of diffusion-weighted MRI scalar maps from FLAIR volumes using generative adversarial networks

**DOI:** 10.3389/fninf.2023.1197330

**Published:** 2023-08-02

**Authors:** Karissa Chan, Pejman Jabehdar Maralani, Alan R. Moody, April Khademi

**Affiliations:** ^1^Electrical, Computer and Biomedical Engineering Department, Toronto Metropolitan University, Toronto, ON, Canada; ^2^Institute of Biomedical Engineering, Science and Technology (iBEST), Toronto, ON, Canada; ^3^Department of Medical Imaging, Sunnybrook Health Sciences Centre, University of Toronto, Toronto, ON, Canada; ^4^Keenan Research Center, St. Michael’s Hospital, Toronto, ON, Canada

**Keywords:** GANs, image synthesis, translation, FLAIR, CycleGAN, pix2pix, DWI

## Abstract

**Introduction:**

Acquisition and pre-processing pipelines for diffusion-weighted imaging (DWI) volumes are resource- and time-consuming. Generating synthetic DWI scalar maps from commonly acquired brain MRI sequences such as fluid-attenuated inversion recovery (FLAIR) could be useful for supplementing datasets. In this work we design and compare GAN-based image translation models for generating DWI scalar maps from FLAIR MRI for the first time.

**Methods:**

We evaluate a pix2pix model, two modified CycleGANs using paired and unpaired data, and a convolutional autoencoder in synthesizing DWI fractional anisotropy (FA) and mean diffusivity (MD) from whole FLAIR volumes. In total, 420 FLAIR and DWI volumes (11,957 images) from multi-center dementia and vascular disease cohorts were used for training/testing. Generated images were evaluated using two groups of metrics: (1) human perception metrics including peak signal-to-noise ratio (PSNR) and structural similarity (SSIM), (2) structural metrics including a newly proposed histogram similarity (Hist-KL) metric and mean squared error (MSE).

**Results:**

Pix2pix demonstrated the best performance both quantitatively and qualitatively with mean PSNR, SSIM, and MSE metrics of 23.41 dB, 0.8, 0.004, respectively for MD generation, and 24.05 dB, 0.78, 0.004, respectively for FA generation. The new histogram similarity metric demonstrated sensitivity to differences in fine details between generated and real images with mean pix2pix MD and FA Hist-KL metrics of 11.73 and 3.74, respectively. Detailed analysis of clinically relevant regions of white matter (WM) and gray matter (GM) in the pix2pix images also showed strong significant (*p* < 0.001) correlations between real and synthetic FA values in both tissue types (*R* = 0.714 for GM, *R* = 0.877 for WM).

**Discussion/conclusion:**

Our results show that pix2pix’s FA and MD models had significantly better structural similarity of tissue structures and fine details than other models, including WM tracts and CSF spaces, between real and generated images. Regional analysis of synthetic volumes showed that synthetic DWI images can not only be used to supplement clinical datasets, but demonstrates potential utility in bypassing or correcting registration in data pre-processing.

## 1. Introduction

Scalar maps such as mean diffusivity (MD) and fractional anisotropy (FA) are typically derived from diffusion weighted MRI (DWI), and used as proxies of water diffusion and diffusion directionality in brain tissue, respectively. Increased water diffusion and decreased directionality are related to microstructural tissue integrity loss related to neurodegenerative diseases such as dementia. However, acquiring scalar maps from DWI relies on processing pipelines such as Tractoflow (Theaud et al., 2020), which are computationally expensive, time-consuming, and susceptible to errors. Additionally, retrospective datasets may not have DWI, which limits analysis. To overcome these challenges, this work investigates generative adversarial networks (GANs) to generate DWI scalar maps from fluid-attenuated inversion recovery (FLAIR) MRI. The FLAIR sequence suppresses signal from cerebrospinal fluid (CSF) and highlights white matter disease and white matter lesions (WML) and as a result, FLAIR images are commonly acquired in both clinical and research contexts. Furthermore, existing works have found correlations between FLAIR intensity, volume, and texture biomarkers and DWI FA and MD measures in whole-brain, gray matter, and white matter regions (Bahsoun et al., 2022; Chan et al., 2023). Since FLAIR MRI can easily be acquired and has established biomarker relationships with FA and MD metrics, it is a good candidate for image synthesis of MD and FA maps. Synthetic data can augment clinical datasets in segmentation and classification tasks (Conte et al., 2021; Sajjad et al., 2021; Platscher et al., 2022).

Recent studies successfully used generative models for translation between brain MRI modalities to synthesize mainly between T1 and T2-weighted images (Kazuhiro et al., 2018; Plassard et al., 2018; Welander et al., 2018; Yang et al., 2018; Chong and Ho, 2021; Osman and Tamam, 2022; Zhan et al., 2022; Zhang et al., 2022). Only few works generated synthetic DWI scalar maps from T1-weighted images (Gu et al., 2019; Hirte et al., 2021), while none have conducted experiments using the FLAIR modality. In this work, we design and evaluate GAN-based image translation models to synthesize DWI maps from whole volume FLAIR MRI for the first time. In addition to traditional CycleGAN and pix2pix models, which are the most commonly used architectures for medical image generation (Kazeminia et al., 2020; Lan et al., 2020; Gong et al., 2021; Jeong et al., 2022; Shokraei Fard et al., 2022; Skandarani et al., 2023), we design a CycleGAN model that includes spectral normalization layers and Gaussian noise in the discriminators to combat mode collapse due to the diversity of training slices from entire volumes. We use label smoothing in the discriminator loss functions, and different initial learning rates for the generators and discriminators. For ablation analysis, we also compare the GAN-based models to a standard autoencoder architecture.

We make several contributions. First, we design and develop the first image translation tools for synthesizing DWI scalar maps from FLAIR MRI. Second, while previous works used only several slices from each volume for training and generating synthetic data, we demonstrate anatomical diversity in our GAN models with the use of full volumes. Third, we integrate the Frechet Inception Distance (FID) into training to reduce resource consumption and the FID is computed using a state-of-the-art medical imaging pre-trained architecture. Lastly, we propose a new performance metric based on histogram KL divergence to evaluate the quality of structural information in the generated images. Commonly used GAN evaluation metrics including PSNR and SSIM are criticized for instability and insensitivity shortcomings in medical imaging (Wang et al., 2004; Huynh-Thu and Ghanbari, 2008; Pambrun and Noumeir, 2015). We hypothesize the new metric can measure subtle local differences between generated and real images. We also perform regional analyses of the WM and GM to investigate the quality of synthetic structural tissue regions.

## 2. Materials and methods

### 2.1. Data

Two datasets of brain FLAIR and DWI MRI are used in this work. The first is from the Canadian Consortium on Neurodegeneration in Aging (CCNA) (Mohaddes et al., 2018) which consists of 313 DWI volumes (9,012 images) with corresponding FLAIR MRI volumes. The dataset is a large dementia cohort which includes subjects diagnosed with mild cognitive impairment, Alzheimer’s disease, vascular dementia, Mixed etiology, and healthy elderly patients. The second is the Canadian Atherosclerosis Imaging Network (CAIN) (Tardif et al., 2013) dataset, which consists of 107 DWI volumes (2,989 images) and corresponding FLAIR MRI volumes of subjects with cerebrovascular disease. Acquisition parameters of the FLAIR and DWI volumes from each dataset are summarized in Table 1.

**TABLE 1:** FLAIR and dMRI acquisition parameters for CCNA and CAIN datasets.

Dataset	Modality	TR (s)	TE (s)	TI (s)	X/Y Spacing (mm)	Z (mm)	Directions
CCNA	FLAIR	9–9.84	0.12–0.146	2.25–2.5	0.9375	3	–
	dMRI	6.9–13	0.064–0.101	–	0.9375–2.6506	2–3	31–33
CAIN	FLAIR	9–11	0.117–0.148	2.2–2.8	0.428–1	3	–
	dMRI	8.8	0.076–0.083	–	1.484–2.969	3–3.6	25–27

### 2.2. Pre-processing and sampling

All FLAIR volumes were brain extracted (DiGregorio et al., 2021) and intensity normalized (Reiche et al., 2019). The ground truth MD and FA volumes were extracted from the DWI volumes using Tractoflow along with corresponding WM and GM masks segmented in Tractoflow (Theaud et al., 2020). The MD and FA volumes and masks were co-registered with the FLAIR volumes to the Brainder FLAIR atlas (Winkler et al., 2009) with dimensions of 256 × 256 × 55. Training and test data were sampled with 80/20 splits. Slices with at least 15% tissue relative to background were used for training to avoid instability in model training caused by slices with little/no brain tissue. This resulted in a total of 9,305 training (327 patients) and 2,396 test images (84 patients) for each modality for the paired data. For unpaired training, a paired test set of 42 patients (1,460 images) was held out to evaluate model performances against ground truths. The remaining 378 patients were randomly split in half to ensure FLAIR and DWI training images came from different patients, resulting in two sets of 6,277 training images. All images are normalized between −1 and 1. To compute the FID during training, 256 images from 9 subjects from the training set were held out.

### 2.3. Deep learning models

**FIGURE 1:**
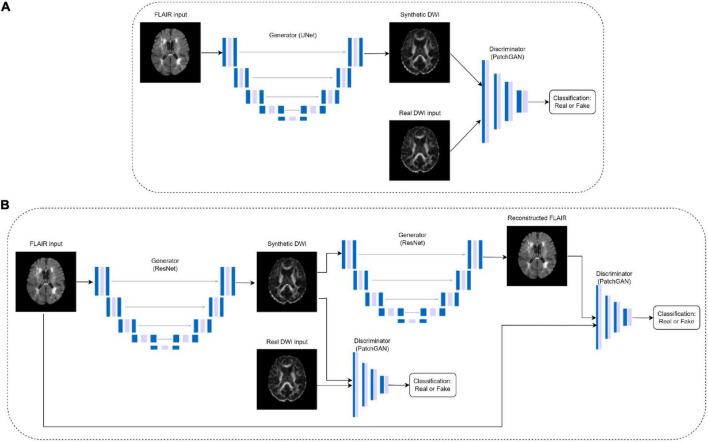
Pix2pix **(A)** and CycleGAN **(B)** architectures, where the DWI images are either FA or MD. The CycleGAN architecture is the same for paired and unpaired models, but the DWI inputs in the unpaired models do not match the FLAIR inputs.

To generate synthetic DWI scalar maps using GAN-based translation models, we implement an optimized (paired) CycleGAN model, an unpaired CycleGAN, a paired pix2pix and a standard autoencoder for comparison purposes. Paired indicates images from the two domains are matching, in this case, the registered FLAIR and DWI. CycleGAN was chosen as the base model due to its success in image translation between modalities (Zhu et al., 2017). It employs two generators and two discriminators to learn the forward and inverse mappings between both modalities as shown in Figure 1B. The generators are ResNet encoder-decoder models while the discriminators are convolutional PatchGAN classifiers which classify images as either real or fake.

Preliminary tests on paired CycleGAN showed the baseline model had partial mode collapse in early epochs of training as generators failed to map the diverse anatomical features and instead generated the same anatomy on every slice (Supplementary Figure 1). Mode collapse occurs when the generator cannot output diverse sets of data, but instead finds a certain type of data that continuously fools the discriminator causing the entire network to over-optimize on one type of data being generated. To combat this, spectral normalization was added to each convolutional layer in the discriminator architecture, as in the SN-GAN (Miyato et al., 2018). The authors concluded that spectral normalization is effective at stabilizing GAN training by normalizing the weight matrices in the convolutional layers to constrain the Lipschitz constant, which mitigates exploding gradient and mode collapse problems (Miyato et al., 2018). Additionally, discriminator losses were observed in preliminary tests to converge much faster than the generator losses, which is also indicative of mode collapse. Therefore, Gaussian noise was added to the beginning of each discriminator layer and label smoothing was applied to the discriminator loss function. The modified discriminator architecture is shown in Supplementary Table 1.

An unpaired CycleGAN was trained for comparison purposes, as well as a baseline pix2pix architecture developed by Isola et al. (2018) which does not have cycle consistency loss. The pix2pix model consists of a U-Net generator and a PatchGAN discriminator (Figure 1A). For the GAN models, the generators and discriminators were assigned different initial learning rates of 4e-4 and 1e-4, respectively due to the fast discriminator convergence. Learning rates were fixed for the first half of training, then decayed linearly to zero (Zhu et al., 2017). Additionally, a convolutional autoencoder matching the hidden layers of the pix2pix generator architecture but without skip connections was trained on paired data to examine the performance of the pix2pix discriminator. The architecture details of the autoencoder can be found in Supplementary Table 2. All models were trained for 100 epochs using Adam optimizers. The autoencoder and pix2pix models used a batch size of 8, while the two CycleGAN models used a batch size of 1. The autoencoder used a learning rate of 4e-4. All experiments were performed using a NVIDIA V100 Volta GPU with 32G HBM2 memory and implemented in Python 3.8 using Tensorflow 2.10.

### 2.4. Loss functions

CycleGAN uses adversarial loss and cycle consistency loss (Zhu et al., 2017), where the adversarial loss (Eq. 1) matches the distribution of the generated images to the targets, and cycle consistency loss (Eq. 2) allows the model to learn forward and backward mappings between the two domains. The least square error is used for the adversarial loss, which yields more stable performances in CycleGAN (Zhu et al., 2017). The total objective is the sum of two adversarial losses, one for each generator, and one cyclic loss (Eq. 3).


(1) $$L_{G\,A\,N}\,\left(G,D_{Y},X,Y\right)=E_{y}∼p_{d\,a\,t\,a}\,\left(y\right)\,\left[{\left(D_{Y}\,\left(y\right)-1\right)}^{2}\right]$$

$$+E_{x}∼p_{d\,a\,t\,a}\,\left(x\right)\,\left[D_{Y}\,{\left(G\,\left(x\right)\right)}^{2}\right]$$

(2) $$L_{c\,y\,c}\,\left(G,F\right)=E_{x}∼p_{d\,a\,t\,a}\,\left(x\right)\,\left[||F\,\left(G\,\left(x\right)\right)-x||\right]$$

$$+E_{y}∼p_{d\,a\,t\,a}\,\left(y\right)\,\left[||G\,\left(F\,\left(y\right)\right)-y||\right]$$

(3) $$L\,\left(G,F,D_{X},D_{Y}\right)=L_{G\,A\,N}\,\left(G,D_{Y},X,Y\right)$$

$$+L_{G\,A\,N}\,\left(F,D_{X},Y,X\right)+\lambda \,L_{c\,y\,c}\,\left(G,F\right)$$

The pix2pix model was trained using binary cross entropy (BCE) for both generator and discriminator losses (Isola et al., 2018). The total pix2pix generator loss is the combination of L1 loss, which is the mean absolute error (MAE) between generator output and target, and adversarial loss (BCE loss of discriminator output). The total discriminator loss is the combination of real and generated BCE losses. The pix2pix generator and discriminator losses are shown in Equations 4 and 5, respectively. The autoencoder was trained with MSE loss.


(4) $$L_{G}\,\left(G,D,X,Y\right)=B\,C\,E\,\left(D\,\left(y\right)\right)+\lambda \,M\,A\,E\,\left(Y,G\,\left(x\right)\right)$$

Where λ = 100 as defined in Isola et al. (2018).


(5) $$L_{D}\,\left(G,X,Y\right)=B\,C\,E\,\left(Y\right)+B\,C\,E\,\left(G\,\left(x\right)\right)$$

### 2.5. Evaluation metrics

Two groups of evaluation metrics, based on human perception and structural information, are used to evaluate the generated test images. The human perception metrics include peak signal-to-noise ratio (PSNR) (Horé and Ziou, 2010) and structural similarity index (SSIM) (Conte et al., 2021). These are shown in Equations 6 and 7.


(6) $$P\,S\,N\,R=10\,\log_{10}\frac{{\left(L-1\right)}^{2}}{M\,S\,E}$$

(7) $$SSIM\left(x,y\right)={\left[l\left(x,y\right)\right]}^{\propto }\cdot [{\left(c\left(x,y\right)\right]}^{\beta }\cdot {\left[s\left(x,y\right)\right]}^{\gamma }$$

Where *l*, *c*, and *s* are the three components of luminance, contrast, and structure and ∝, β, γ are parameters for adjusting the weight of each component:


$$l\,\left(x,y\right)=\,\frac{2\,\left(1+R\right)}{1+{\left(1+R\right)}^{2}+\frac{C_{1}}{\mu_{x}^{2}}},c\,\left(x,y\right)=\frac{\left(2\,\sigma_{x}\,\sigma_{y}+C_{2}\right)}{\left(\sigma_{x}^{2}+\sigma_{y}^{2}+C_{2}\right)},$$

$$s\,\left(x,y\right)=\frac{\left(\sigma_{x\,y}+C_{3}\right)}{\left(\sigma_{x}+\sigma_{y}+C_{3}\right)}$$

where *x* and *y* are two images being compared, *R* is the size of luminance change relative to background luminance, μ is the mean intensity of an image, σ is the standard deviation of an image, and *C*_1,2,3_ are constants.

The structural metrics include mean squared error (MSE) and a proposed metric measuring the KL divergence of histograms (Hist-KL) between real and generated images. The histogram of an image reflects the probability distribution of the pixels within the image. Histogram analysis is particularly important when evaluating modalities such as DWI in which different tissue types appear at different intensities, thus corresponding to specific histogram peaks. Additionally, previous studies have found that increased kurtosis of FLAIR histogram distributions is related to worsening cognition and decreased tissue integrity (Bahsoun et al., 2022), demonstrating that changes in tissue are reflected in the histogram. Therefore, we hypothesize that differences related to tissue structures in the real vs. generated images can be assessed by measuring the distance between their histograms. Low Hist-KL indicates high degree of similarity between images. The MSE and Hist-KL computations are shown in Equations 8 and 9.


(8) $$M\,S\,E=\frac{1}{m\,n}\,\sum_{i=0}^{m-1}\sum_{j=0}^{n-1}{\left(x\,\left(i,j\right)-y\,\left(i,j\right)\right)}^{2}$$

(9) $$HistKL=KL\left(P||Q\right)=-\sum_{x}P\left(X\right)\log\frac{P\,\left(X\right)}{Q\,\left(X\right)}$$

Where *P* and *Q* are the distributions of the two images, and *x* is the histogram bins.

### 2.6. Frechet Inception Distance (FID)

The FID score is commonly used for evaluating GAN performance (Heusel et al., 2018). It employs a pre-trained classification model, InceptionV3, to generate feature vectors of real and fake images, then quantifies similarity between images by measuring the difference between feature vectors. However, InceptionV3 is pretrained on natural images from ImageNet, which may be non-optimal for medical imaging applications. In this work, FID was computed using an InceptionV3 model pre-trained on a large medical imaging dataset called RadImageNet (Rad-InceptionV3) containing 1.35 million annotated medical images with 3 modalities, 11 anatomies, and 165 pathologies (Mei et al., 2022). Typically, the FID is computed after training to determine the best epoch and requires a massive sample size of at least 50,000 images to obtain reliable values. To reduce resource consumption, we implemented the FID score during model training. Computing FID during training requires only a small sample size (256) of generated images with a large number ( > > 2,048) of real images (Mathiasen and Hvilshøj, 2021). All 9,305 real images in the training set and 256 fake images generated from the held out validation set were used to compute the FID score at the end of each epoch. For all GANs, lowest FID score was used to select the best epoch. Additional analyses on the FID computation during training and optimal epoch selection can be found in section 2 of the Supplementary Material Data Sheet.

## 3. Results

### 3.1. Model performances

**FIGURE 2:**
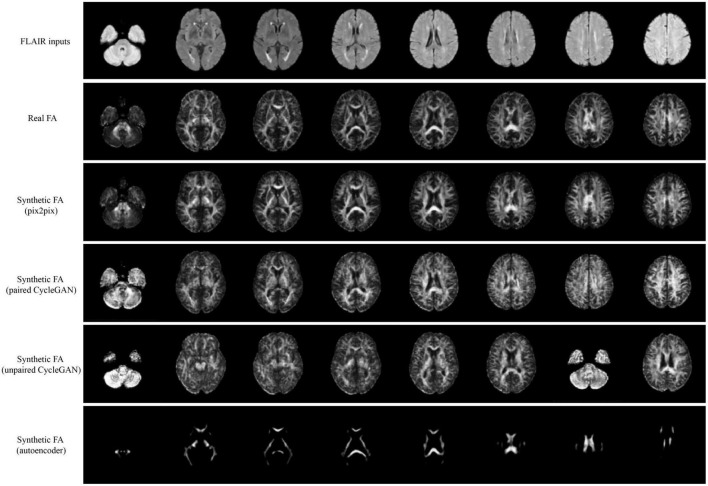
Samples of FLAIR inputs (top row), real FA (second row) and generated FA slices from each model. For paired models, the images shown are different slices from the same patient volume. From left to right, randomly sampled lower to upper slices (12 to 35) are shown.

**FIGURE 3:**
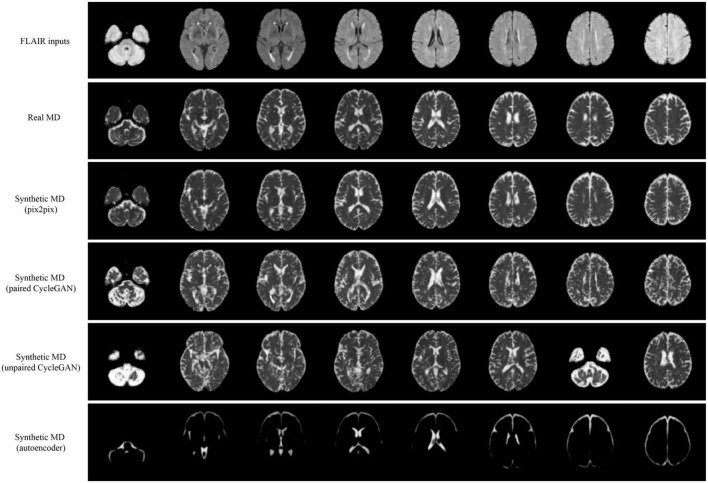
Samples of FLAIR inputs (top row), real MD (second row) and generated MD slices from each model. For paired models, the images shown are different slices from the same patient volume. From left to right, randomly sampled lower to upper slices (12 to 35) are shown.

Qualitative results for all models are shown in Figures 2, 3. The pix2pix model generates images with high visual correspondence with the ground truth, and seems to best capture complex anatomy such as WM tracts in FA and CSF regions in MD. The optimized paired CycleGAN has good correspondence, but variability and inability to resolve fine-details are noted in the unpaired CycleGAN. The autoencoder fails to generate anything sensible.

**FIGURE 4:**
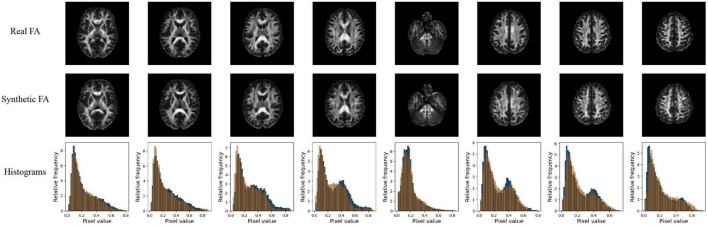
Pix2pix FA generation. **Top row**: real FA, **middle row**: generated FA, **bottom row**: FA histograms (real is blue, generated is orange). All slices shown belong to the same patient volume and are randomly sampled between slices 16 (lower) to 40 (upper).

**FIGURE 5:**
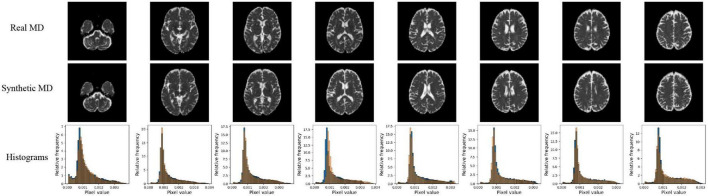
Pix2pix MD generation. **Top row**: real MD, **middle row**: generated MD, **bottom row**: MD histograms (real is blue, generated is orange). All slices shown belong to the same patient volume and are randomly sampled between slices 16 (lower) to 40 (upper).

Quantitative performance for MD and FA models is shown in Table 2. The distribution of the metric, with *t*-tests between models is shown in the Supplementary Figure 2. The pix2pix model performed the best across all metrics, for both MD and FA (*p <* 0.05). Figures 4, 5 show several pix2pix generations for MD and FA along with the corresponding histograms. There is high visual similarity between generations and real images which is exemplified by the histograms. The PSNR and SSIM results for the pix2pix in this work for MD and FA images are comparable to existing literature as shown in Table 3. CycleGAN models performed worse than pix2pix but the paired CycleGAN demonstrated better performance across all metrics than the unpaired, which corresponds to the qualitative findings. The autoencoder performed poorly and was not considered further.

**TABLE 2:** Mean (standard deviation) of evaluation metrics over all models.

Model	PSNR in dB ↑	SSIM ↑	Hist-KL ↓	MSE ↓
MD pix2pix	**23.41 (1.9)**	**0.80 (0.044)**	**11.73 (16.7)**	**0.005 (0.002)**
MD CycleGAN (paired)	19.73 (1.77)	0.73 (0.047)	27.43 (20.9)	0.011 (0.004)
MD CycleGAN (unpaired)	19.03 (2.90)	0.72 (0.10)	34.92 (24.6)	0.013 (0.0068)
MD Autoencoder	16.44 (1.53)	0.63 (0.073)	79.11 (27.5)	0.024 (0.0069)
FA pix2pix	**24.0 (1.16)**	**0.78 (0.033)**	**3.74 (3.15)**	**0.004 (0.001)**
FA CycleGAN (paired)	19.26 (2.76)	0.71 (0.044)	26.46 (23.4)	0.014 (0.009)
FA CycleGAN (unpaired)	19.04 (1.80)	0.69 (0.034)	28.32 (18.2)	0.014 (0.006)
FA Autoencoder	18.30 (1.88)	0.64 (0.069)	71.44 (38.5)	0.016 (0.007)

Bold indicates best performance of MD and FA models. Metrics with ↑ indicate better performance with a larger value, and metrics with ↓ indicate better performance with a smaller value.

**TABLE 3:** Summary of studies involving image-to-image translation of brain MRI modalities.

Model	Input/Output modalities	Performance
CycleGAN (Gu et al., 2019)	T1w/DWI scalars	SSIM: 0.861 (MD) SSIM: 0.948 (FA)
introVAE, StyleGAN (Hirte et al., 2021)	T1w/DWI	Comparable ISD and LVSS to real dataset
CycleGAN (paired) (Welander et al., 2018)	T1w/T2w	PSNR: 24 (T1w) PSNR: 24.15 (T2w)
Switchable CycleGAN (paired) (Zhang et al., 2022)	T1w/T2w	PSNR: 31.733, SSIM: 0.723 (T1w) PSNR: 31.671, SSIM: 0.747 (T2w)
MGM-GAN (Zhan et al., 2022)	T1 + T1c + T2/FLAIR	PSNR: 26.801, SSIM: 0.918
U-Net (Osman and Tamam, 2022)	T1/FLAIR	PSNR: 33.25, SSIM: 0.946
cGAN+L1 (Yang et al., 2018)	T1/T2	PSNR: 29.979, SSIM: 0.69
pix2pix (this work)	FLAIR/DWI scalars	PSNR: 23.41, SSIM: 0.80, Hist-KL: 11.73 (MD) PSNR: 24.05, SSIM: 0.78, Hist-KL: 3.74 (FA)

**FIGURE 6:**
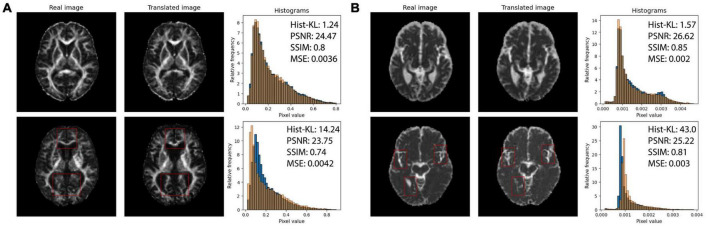
**(A)** Real (first column) and pix2pix generated (second column) FA images of middle slices with low Hist-KL (top row) and high Hist-KL (bottom row). Corresponding histograms of real (blue) and generated (orange) images are shown in the third column. **(B)** Real (first column) and pix2pix generated (second column) MD images with low Hist-KL (top row) and high Hist-KL (bottom row). Corresponding histograms of real (blue) and generated (orange) images are shown in the third column. All slices shown belong to different patients. Notable visual differences between real and synthetic images in the high Hist-KL images are denoted with red boxes.

Structural similarity index and PSNR may not be adequately quantifying the subtle differences between generated and real images, such as overestimating CSF in the sulci and gyri (subarachnoid spaces) and underestimation of small structures such as WM tracts. The fine-details and structural similarity between generated and real images may be better measured by Hist-KL. See Figure 6 for pix2pix FA and MD images with high and low Hist-KL values. The PSNR and SSIM values between high and low Hist-KL images are similar, while there is a large difference in their Hist-KL values. Structures in the generated images are more anatomically accurate and aligned for low Hist-KL images (see WM tracts in FA and CSF spaces in MD) which is highlighted by the histograms. For generated images with large histogram differences (high Hist-KL), images have vastly different histograms, which is representative of the local, subtle spatial inaccuracies of the method. Thus, this metric may be more sensitive to differences in fine-details and microstructure than the standard visual perception metrics, making it useful in image generation and super resolution particularly for medical imaging.

### 3.2. Regional analysis of synthetic volumes

The best-performing pix2pix method was further evaluated in its ability to synthesize accurate structural information by regional analysis. All further experiments use only pix2pix generated test volumes.

#### 3.2.1. Performance metrics in GM and WM regions

**FIGURE 7:**
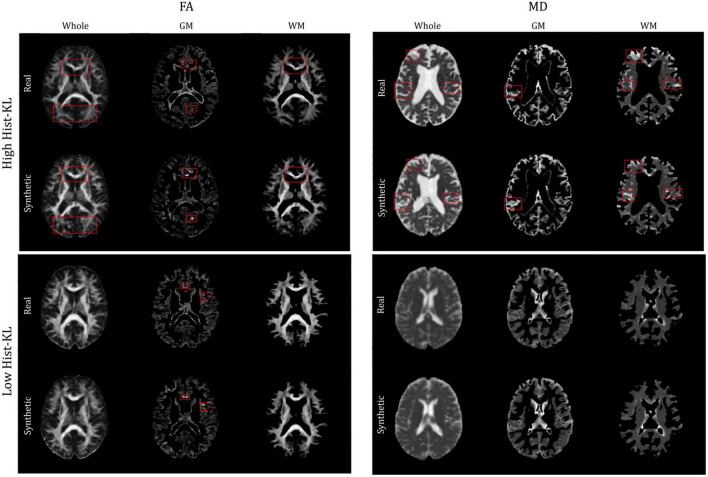
Sample FA (left column) and MD (right column) images of middle slices with segmented GM and WM regions of different patients with high Hist-KL (top row) and low Hist-KL (bottom row). In high Hist-KL volumes, the most notable visual differences are outlined in red boxes. In the low Hist-KL volumes, the GM and WM structures are very similar between real and synthetic with only some visual differences.

The gray and white matter tissue regions of the MD and FA test volumes were analyzed. From the test set, a total of 32 patients from both datasets had corresponding registered GM and WM masks. The masks were used to segment GM and WM from both real and synthetic volumes, and evaluation metrics were computed in the sub-regions. Table 4 shows the mean evaluation metrics for GM and WM. All metrics were better in FA for the WM compared to the GM region, which indicates excellent reconstruction of the WM tracts. In MD the findings were similar when considering PSNR and SSIM, however, upon inspection of the Hist-KL metric there are discrepancies, which further strengthens our hypothesis that Hist-KL provides valuable information on image synthesis. The high value for the MD Hist-KL metric in the WM regions can be attributed to both under- and over-estimation of CSF in the synthetic MD volumes, particularly in volumes with large amounts of CSF (Figure 7, top right). In comparison, MD volumes with low Hist-KL (Figure 7, bottom right) demonstrate inherently less CSF, as seen in the smaller amount of hyperintensities along the edges of the brain. As the amount of CSF in MD varies drastically between patients, it is expected that perfectly accurate CSF regions will be difficult to synthesize thus resulting in higher Hist-KL metrics overall in MD relative to FA. Figure 7 (left column) also shows sample FA from GM and WM regions of the subjects with highest and lowest Hist-KL metrics. The volumes with low Hist-KL metrics have very similar real and synthetic WM and GM structures with little to no overlap of tissues between regions after masking. Additionally, the synthetic GM have fewer border artifacts from registration than the real images. In high Hist-KL volumes, the main cause of histogram KL divergence is the warping of the WM tracts in the real registered images, while they are generated properly in the synthetic images. This suggests that synthetic data generation may be used to reduce registration errors.

**TABLE 4:** Mean metrics (with SD) computed across all MD and FA test volumes.

	MD				FA			
	PSNR	SSIM	Hist-KL	MSE	PSNR	SSIM	Hist-KL	MSE
**GM**	27.69 (1.17)	0.955 (0.008)	**4.71 (1.68)**	0.002 (0.0005)	28.89 (0.92)	0.927 (0.012)	3.56 (1.43)	0.0015 (0.0003)
**WM**	**30.81 (2.06)**	**0.966 (0.013)**	6.04 (2.32)	**0.0011 (0.0005)**	**29.6 (1.04)**	**0.951 (0.011)**	**2.14 (0.93)**	**0.0014 (0.0004)**

Bold values indicate best performance metric.

**FIGURE 8:**
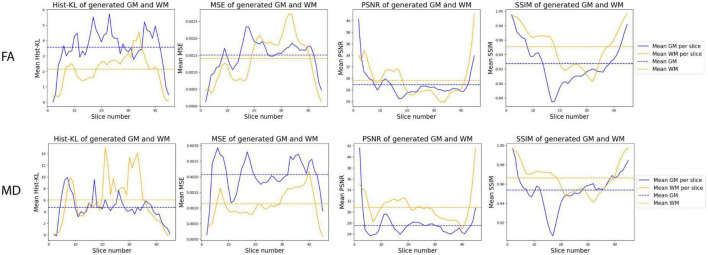
Distribution of mean metrics per slice across all synthetic FA **(top row)** and MD **(bottom row)** test volumes.

**FIGURE 9:**
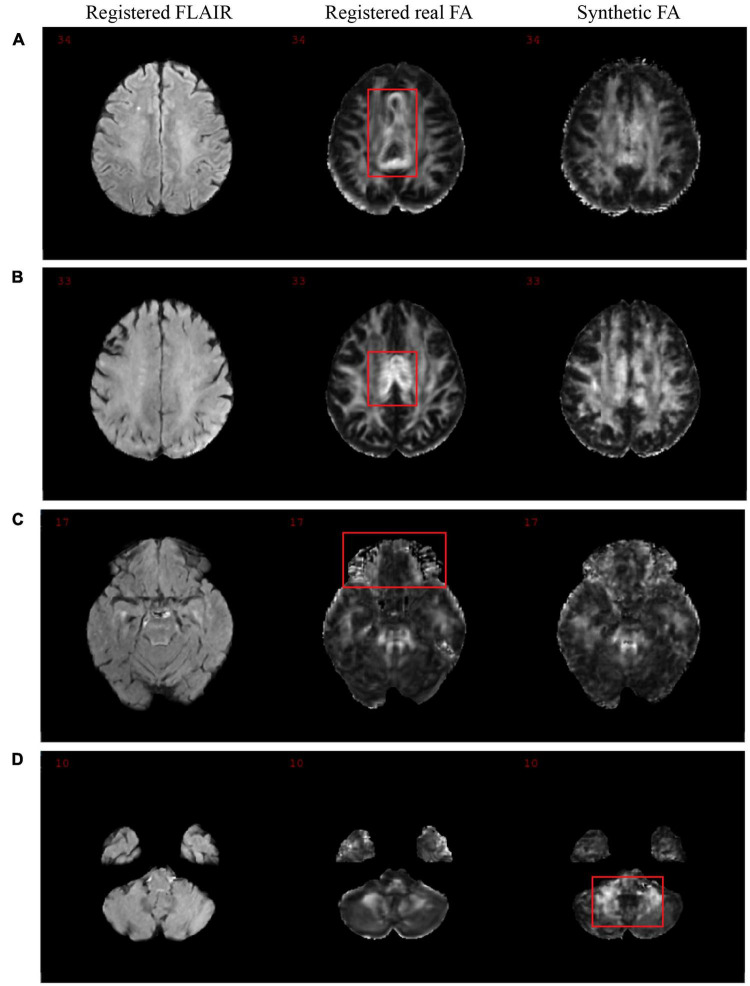
Samples of real and synthetic upper slices 33 and 34 **(A,B)**, a lower slice 17 **(C)**, and cerebellum slice 10 **(D)** from different patients. Registration warping and artifacts in the real images are highlighted in the red boxes in panels **(A–C)**, while the hyperintense errors in the cerebellum in the synthetic image is highlighted in a red box in panel **(D)**.

Figure 8 shows the mean performance metrics per slice over all synthetic test volumes. In both MD and FA, the PSNR metric is consistent across slices. However, the other metrics show worse performance in the WM region for higher numbered slices, corresponding to the upper (superior) slices of the brain volumes. On the other hand, in the GM region, lower (inferior) slices related to cerebellar structures showed worse performance. Samples of upper and lower slices from different patients show mismatched histograms between real and synthetic FA volumes due to registration warping (Figures 9A, B) and artifacts (Figure 9C) of the real images. This suggests that generating synthetic FA maps using FLAIR images may remove the need for co-registration pipelines and thus reducing registration errors, as the generated images are in the same space as the input images. However, another cause for low GM performance is in the cerebellum of the synthetic images, where the model inaccurately generates hyperintense regions in the synthetic cerebellar GM (Figure 9D).

#### 3.2.2. Correlations between real and synthetic data

**FIGURE 10:**
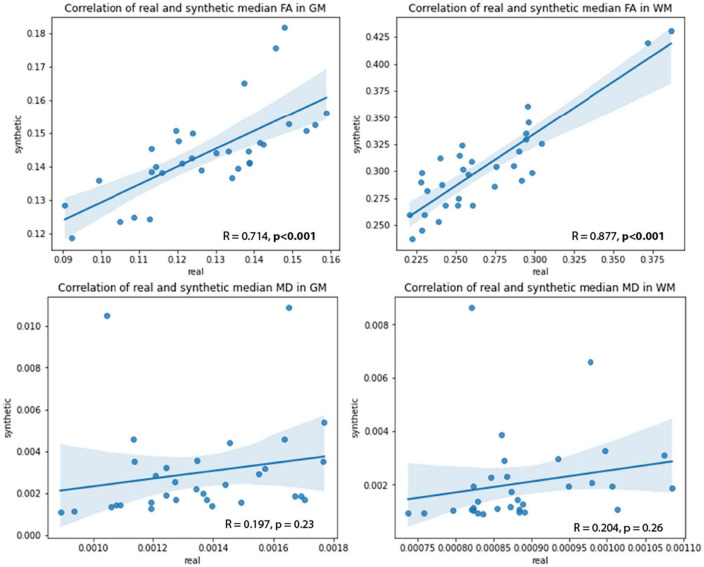
Correlations between real and synthetic FA **(top row)** and MD **(bottom row)** in GM and WM regions.

Pearson’s correlation tests were used to examine the relationships between real and synthetic MD and FA volumes. The median MD and FA values of the GM and WM were extracted from real and synthetic volumes and correlated to one another (Figure 10). Strong and significant (*p* < 0.001) R correlations of 0.71 and 0.88 were found between real and synthetic FA of the GM and WM, respectively, while no significant correlations were found for MD volumes (Table 5). This may be attributed to the overestimation of CSF in synthetic MD as seen in the high Hist-KL example of generated MD shown in Figure 7, resulting in significantly increased median MD values of the synthetic volumes.

**TABLE 5:** R correlation coefficients between real and synthetic MD and FA values.

	MD		FA	
	*R*	*p*	*R*	*p*
**GM**	0.197	0.23	**0.714**	**<0.001**
**WM**	0.204	0.26	**0.877**	**<0.001**

Bold *R*-values indicate strong correlations, and bold *p*-values indicate significant correlations.

## 4. Discussion

In this work, performance of GAN-based image translation tools for synthesizing DWI scalar maps from whole-volume FLAIR MRI, is investigated. Three architectures were investigated including an optimized CycleGAN for paired translation that employs Gaussian noise and spectral normalization to combat mode collapse, a CycleGAN trained with unpaired data, and a pix2pix model (no cycle consistency) with paired data. For ablation purposes, a convolutional autoencoder was also compared, which is essentially pix2pix without the discriminator. FID scores, computed using Rad-InceptionV3 (a novel medical imaging pre-trained network), were used to determine the optimal epoch on the fly rather than after training. Through qualitative and quantitative performance, pix2pix offered the highest quality image generations for MD and FA images. This was followed by the optimized paired CycleGAN, unpaired CycleGAN and lastly, the autoencoder, which failed to generate anything meaningful for the task.

The pix2pix model outperformed all other models. Comparing to the autoencoder, our findings demonstrate the utility of a discriminator network to force outputs to be more realistic. The autoencoder used MSE loss and was unable to reconstruct the complex mappings between FLAIR and DWI. We expected the paired CycleGAN to yield similar results to pix2pix. However, pix2pix was qualitatively and quantitative superior, which may be attributed to pix2pix’s objective function that leverages paired data to learn pixel-wise mappings between modalities. On the other hand, CycleGAN matches FLAIR and DWI domain distributions, but does not directly map each input pixel to the output. The paired CycleGAN performed better than the unpaired model, demonstrating the modifications we proposed to mainly remove mode collapse improved performance.

Perceptual metrics (PSNR and SSIM) from our pix2pix models are comparable to those in existing literature (Welander et al., 2018; Gu et al., 2019; Zhang et al., 2022), which use T1w images to generate DWI and T2w images. Contrasted to previous studies, the FA model resulted in better performance metrics overall than the MD model, with subtle differences noted in the Hist-KL metric, showing GAN-based models are able to generate fine structures such as WM tracts with good resolution and detail. Perceptual metrics have been widely used to evaluate the visual quality of synthetic images, but these metrics may not correlate to accuracy of generating important anatomical structures such as WM tracts or CSF spaces. The proposed Hist-KL metric is useful in this regard, as differences in intensity distributions are related to the number of pixels in each tissue region. Any under- or overestimation of tissue types (such as CSF in MD), are reflected in the histogram and captured by this metric. For the same method, the main contributor to differences in Hist-KL seems to be the varying amount of under/over-estimated tissue present in the real images. As visualized in Figure 7, the real image of the high Hist-KL MD sample has substantially larger amounts of CSF than that of the low Hist-KL sample, which increases metric variability within a particular method and modality. Further, findings from regional analysis demonstrate that the pix2pix model performs better in the WM regions than the GM. The FA scalar offers unique information on microstructural tissue integrity and tractography, thus synthetic FA volumes with anatomically accurate WM structures would be extremely valuable in supplementing datasets. However, further validation of the synthetic FA volumes with respect to performance in WM tract segmentation pipelines is required.

Greater errors between real and synthetic images were mainly attributed to registration warping in the real volumes as seen in Figures 9A, B. The warping in the real images is due to registration errors from co-registering real DWI and FLAIR to FLAIR atlas space. This is a necessary pre-processing step in order to have both volumes in the same space for studies requiring analysis of both modalities. Registration from DWI space to FLAIR space can be difficult as their resolutions in native space can be drastically different. For example, a matched pair of FLAIR and DWI volumes of a CCNA patient have voxel sizes 0.94 × 0.94 × 3 mm and 2.65 × 2.65 × 2 mm, respectively. The DWI volumes have substantially lower in-plane resolution, limiting the detail of fine anatomical structures in the DWI volumes in contrast to FLAIR. As registration requires non-linear deformations, the distortion in the registered DWI volumes is caused by attempting to estimate the deformation fields from low resolutions (less detail) to high resolutions (more detail), and vice versa. A standard solution to minimize distortion is to resample the volumes to similar resolutions before registration; however, this can also cause loss of information or interpolation artifacts when resampling to largely different voxel sizes. As such, our findings demonstrate that synthetic image generation offers a potentially useful method to bypass both registration and resampling (and effectively reducing warping errors) by generating images in the native space of the input image. However, more investigation into registration methods will be required to make a valid comparison between synthetic and registered images. Lastly, strong correlations between real and synthetic median FA in both WM and GM regions indicate that the synthetic volumes are generated with accurate FA values and thus may be used for analyses alongside real data.

This work proposes generation methods for supplementing datasets with synthetic FA and MD measures. Such methods may also be used for generating other microstructure measures related to diffusion-weighted imaging. A previous study investigated the use of GANs for generating synthetic DWI volumes (Hirte et al., 2021), from which metrics quantifying tissue microstructure such as NODDI parameters and ADC maps may also be extracted. A generation method which could synthesize scalar maps representing all DWI microstructural tissue measures would offer a wide translation potential for clinical utility, as it would replace the need for time-consuming DWI scalar map extraction.

To improve the paired CycleGAN model, future work could leverage the strengths of pix2pix (pixel-wise objective function) and our modified CycleGAN models (cycle consistency). The unpaired model may be improved with additional datasets or 3D CycleGAN models. A limitation of the work includes lower performance in cerebellar slices, which may be due to the small amount of tissue (low sample sizes) and varying anatomy which hinders performance. A 3D model may help, or it may be possible to train separate 2D models for each region. Another limitation of the work is the lower performance of the MD models compared to FA. Future work may include optimization methods to optimize models specifically for MD volume generation. To determine clinical utility, future work could include domain-adaptation, dataset-specific models, and using the generated data in classification/segmentation tasks. Further, model performance should be evaluated on out-of-distribution cohorts to evaluate mapping on entirely different datasets. Future investigation into the generation of other DWI measures would also be clinically useful.

## 5. Conclusion

We design and evaluate GAN-based image translation tools for generating MD and FA scalar maps from FLAIR MRI. Pix2pix is the top performer that can best generate fine details such as WM tracts, due to the objective function that leverages paired data to learn pixelwise mappings between modalities. Ablation with an autoencoder (pix2pix without the discriminator) produces insensible results, which highlights that a discriminator is needed to force realism in the generations. The CycleGAN with paired data was successfully optimized to prevent mode collapse, but because CycleGAN aims to match FLAIR and DWI domain distributions, rather than directly mapping each input pixel to the output, the performance is suboptimal. CycleGAN with unpaired data performs the worst and is likely due to the problem space being too large to resolve fine details in the images. We have also shown that Hist-KL is an effective metric in evaluating the accuracy of tissue structures in synthetic images and may be used alongside existing visual quality metrics. Additionally, regional analysis of volumes generated using the pix2pix model demonstrated that synthetic DWI FA volumes may be useful in supplementing clinical datasets and correcting registration errors.

## Data availability statement

The data analyzed in this study is subject to the following licenses/restrictions: The datasets presented in this article are not readily available because of patient privacy and confidentiality. Requests to access these datasets should be directed to https://ccna.loris.ca/ and AK, akhademi@torontomu.ca.

## Author contributions

KC and AK contributed to the conception and design of the study, performed statistical analysis, and wrote the first draft of the manuscript. All authors contributed to manuscript revision.
