# Supplementary material for: Synthesis of diffusion-weighted MRI scalar maps from FLAIR volumes using generative adversarial networks
Source: Front Neuroinform. 2023 Aug 2;17:1197330. doi: 10.3389/fninf.2023.1197330 (PMC10436214; doi:10.3389/fninf.2023.1197330)
Supplement: Supplementary file 1 [file Data_Sheet_1.docx]

Supplementary Material

Synthesis of Diffusion-Weighted MRI Scalar Maps from FLAIR Volumes using Generative Adversarial Networks

Karissa Chan*, Pejman Jahbedar Maralani, Alan R. Moody, April Khademi

*** Correspondence:** Corresponding Author: Karissa.chan@torontomu.ca

# Supplementary Figures and Tables

## Supplementary Figure Legend

**Supplementary Figure 1.** Examples of mode collapse in preliminary testing using baseline CycleGAN. The top two rows of input images are lower slices of the brain in proximity to the cerebellum. The model incorrectly generates ventricles and major white matter tracts on those slices while correctly generating ventricles in the middle slices shown in the bottom two rows.

**Supplementary Figure 2:** Boxplots comparing evaluation metrics from each model. Asterisks indicate significant differences.

**Supplementary Figure 3:** Plots of the FID scores for each epoch during pix2pix, paired CycleGAN, and unpaired CycleGAN training.

## Supplementary Tables

**Supplementary Table 1**: Modified CycleGAN discriminator. Gaussian noise is added before each convolutional layer. Instance normalization and Leaky ReLU activation are applied after each convolutional layer.

| **Layer** | **Filter size** | **Activation size** |
| --- | --- | --- |
| Input | - | 1x256x256 |
| Spectral Norm 2D Conv | 64x4x4, stride 2 | 64x128x128 |
| Spectral Norm 2D Conv | 128x4x4, stride 2 | 128x64x64 |
| Spectral Norm 2D Conv | 256x4x4, stride 2 | 256x32x32 |
| Spectral Norm 2D Conv | 512x4x4 | 512x32x32 |
| Spectral Norm 2D Conv | 1x4x4 | 1x32x32 |

**Supplementary Table 2**: Convolutional autoencoder architecture. Instance normalization and Leaky ReLU activation are applied after each convolutional layer.

| **Layer** | **Filter size** | **Activation size** |
| --- | --- | --- |
| Input | - | 1x256x256 |
| 2D Conv | 64x4x4, stride 2 | 64x128x128 |
| 2D Conv | 128x4x4, stride 2 | 128x64x64 |
| 2 x 2D Conv | 256x4x4, stride 2 | 256x16x16 |
| 3 x 2D Conv | 512x4x4, stride 2 | 512x2x2 |
| Flatten | - | 20148 |
| Dense layer | - | 200x1 |
| Dense layer | - | 2048x1 |
| Reshape | - | 512x2x2 |
| 2 x 2D Conv Transpose | 512x4x4, stride 2 | 512x8x8 |
| 2 x 2D Conv Transpose | 256x4x4, stride 2 | 256x32x32 |
| 2D Conv Transpose | 128x4x4, stride 2 | 128x64x64 |
| 2D Conv Transpose | 64x4x4, stride 2 | 64x128x128 |
| 2D Conv Transpose | 1x4x4, stride 2 | 1x256x256 |
| sigmoid activation | - | - |

# Supplementary Data: FID Implementation

The FID metric was investigated in further detail to determine if the FID score computed during training is an appropriate method of determining the optimal epoch. The FID scores per epoch are shown in Supplementary Figure 3 for the training of each GAN model. The optimal epoch for every model was chosen as the one with the lowest FID score. For the pix2pix MD and FA models, the optimal epochs were 20 and 31 respectively. For the paired CycleGAN MD and FA models, the optimal epochs were 94 and 85 respectively. For the unpaired CycleGAN MD and FA models, the optimal epochs were 91 and 83 respectively. As seen in the plots, the FID scores in the pix2pix training reached their minimums earlier but demonstrated smaller fluctuations than the CycleGAN. Both CycleGANs demonstrated slightly decreasing trends throughout training, but with large fluctuations indicating some training instability. These trends coincide with the complexity of each model, as we expected that the CycleGAN would be more difficult and longer to train due to the nature of its cycle consistency.

For the paired CycleGAN model, the performances of the model parameters at the optimal epoch and the last training epoch (100) were compared using the evaluation metrics from the held out test set. In the MD model, the generated images using model parameters from the optimal epoch yielded SSIM, PSNR, and MSE values not significantly different to the last epoch as determined by t-tests. However, the Hist-KL values from the optimal epoch were significantly lower (p<0.01) than those for the last epoch, demonstrating that a lower training FID score indicates better performance in terms of histogram matching. For the FA model, SSIM, PSNR, and Hist-KL values were significantly lower (p<0.01) for the optimal epoch. This demonstrates that the implementation of FID during training not only decreases resource and time consumption, but is able to describe the performance of the model at each training epoch in terms of histogram and structural similarity between real and generated images. In the future, it may be implemented as a metric to track training progress or be used in early stopping criteria to further decrease resource and time consumption.
