# Supplementary figures and images for: Synthesis of diffusion-weighted MRI scalar maps from FLAIR volumes using generative adversarial networks
Source: Front Neuroinform. 2023 Aug 2;17:1197330. doi: 10.3389/fninf.2023.1197330 (PMC10436214; doi:10.3389/fninf.2023.1197330)

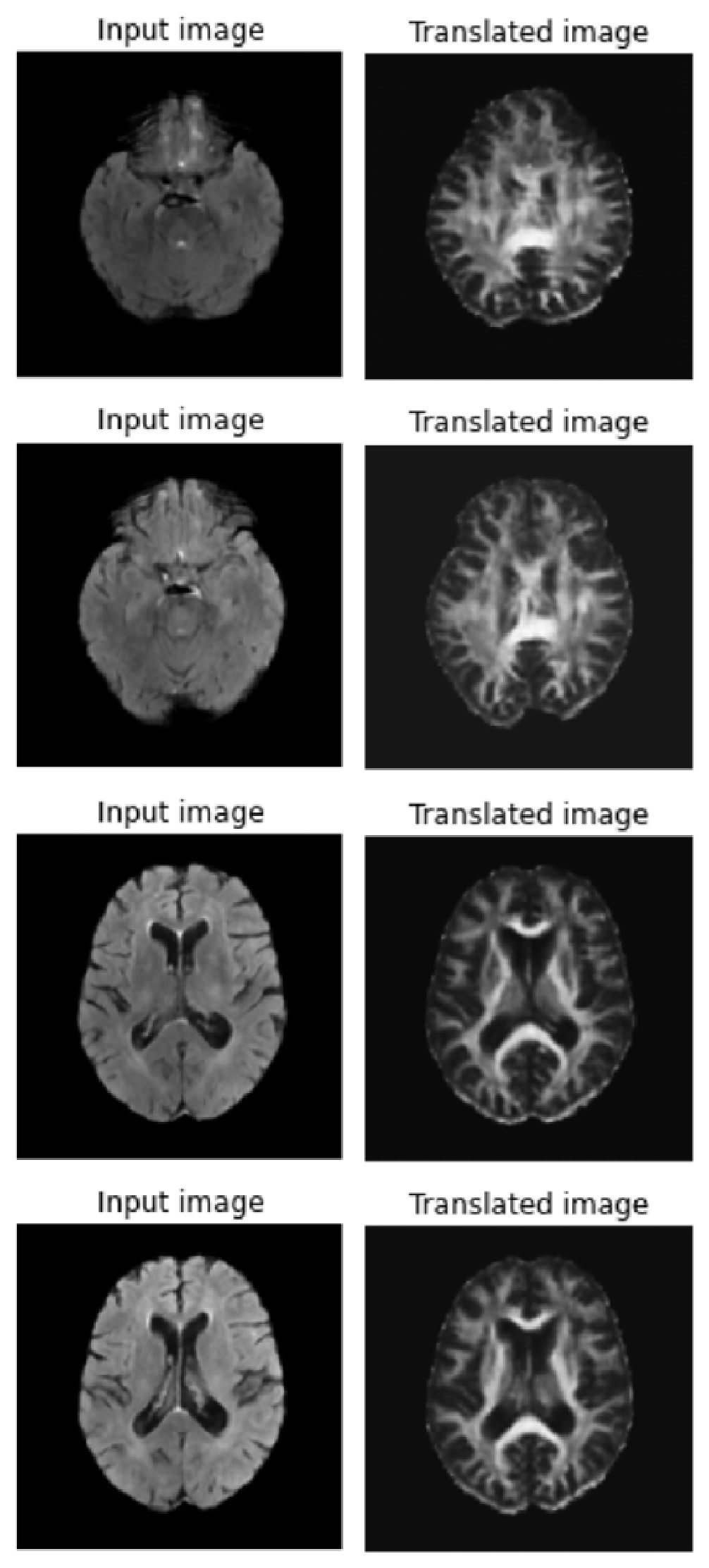

Supplement: Supplementary file 2 [file Image_1.TIF]

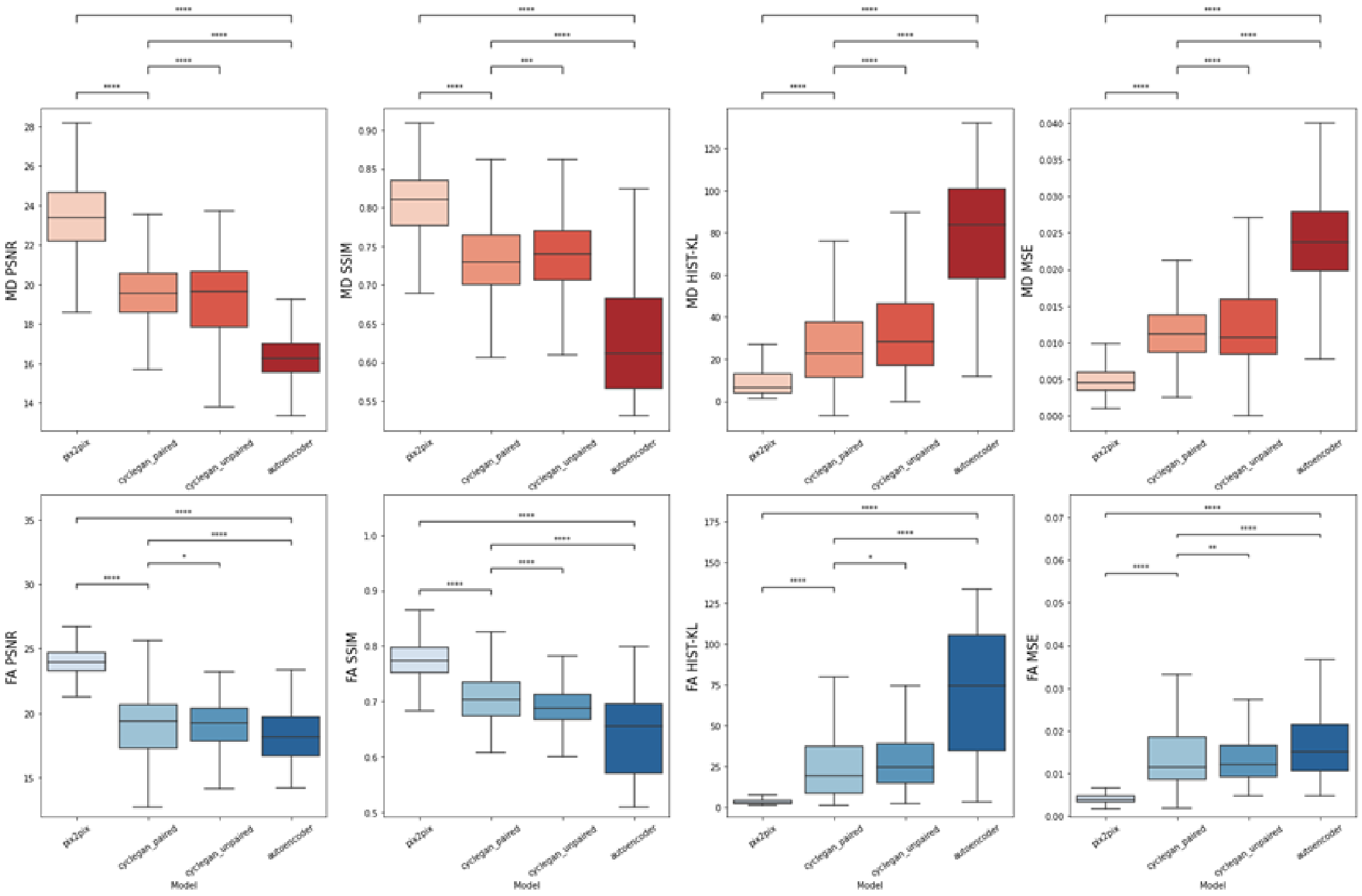

Supplement: Supplementary file 3 [file Image_2.TIF]

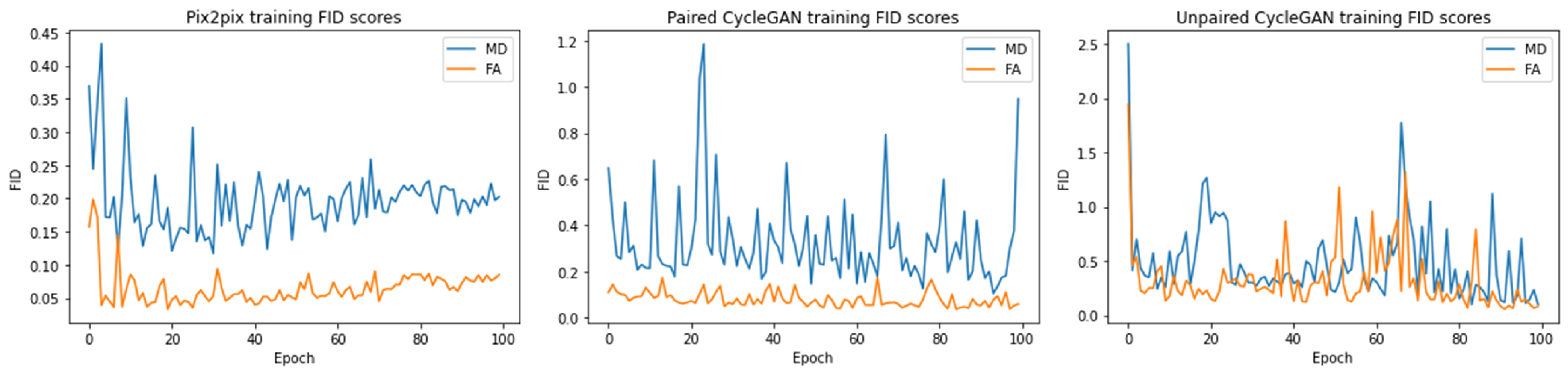

Supplement: Supplementary file 4 [file Image_3.TIF]
